# Shift in the paradigm towards next-generation microbiology

**DOI:** 10.1093/femsle/fnz159

**Published:** 2019-07-17

**Authors:** Blaž Stres, Luka Kronegger

**Affiliations:** 1 Center for Clinical Neurophysiology, Faculty of Medicine, University of Ljubljana, Vrazov trg 2, SI-1000 Ljubljana, Slovenia; 2 Group for Microbiology and Microbial Biotechnology, Department of Animal Science, Biotechnical Faculty, University of Ljubljana, Jamnikarjeva 101, SI-1000 Ljubljana, Slovenia; 3 Institute of Sanitary Engineering, Faculty of Civil and Geodetic Engineering, University of Ljubljana, Hajdrihova 28, SI-1000 Ljubljana, Slovenia; 4 Faculty of Social Sciences, University of Ljubljana, Kardeljeva ploščad 5, SI-1000 Ljubljana, Slovenia

**Keywords:** bibliometric analysis, top-down, publication bias, meta-analyses, bioinformatics, biology, statistics, metagenomics, metaproteomics, metatranscriptomics, metabolomics

## Abstract

In this work, the position of contemporary microbiology is considered from the perspective of scientific success, and a list of historical points and lessons learned from the fields of medical microbiology, microbial ecology and systems biology is presented. In addition, patterns in the development of top-down research topics that emerged over time as well as overlapping ideas and personnel, which are the first signs of trans-domain research activities in the fields of metagenomics, metaproteomics, metatranscriptomics and metabolomics, are explored through analysis of the publication networks of 28 654 papers using the computer programme Pajek. The current state of affairs is defined, and the need for meta-analyses to leverage publication biases in the field of microbiology is put forward as a very important emerging field of microbiology, especially since microbiology is progressively dealing with multi-scale systems. Consequently, the need for cross-fertilisation with other fields/disciplines instead of ‘more microbiology’ is needed to advance the field of microbiology as such. The reader is directed to consider how novel technologies, the introduction of big data approaches and artificial intelligence have transformed microbiology into a multi-scale field and initiated a shift away from its history of mostly manual work and towards a largely technology-, data- and statistics-driven discipline that is often coupled with automation and modelling.

## INTRODUCTION: A FEW HISTORICAL POINTS TO CONSIDER REGARDING SUCCESS IN SCIENCE

The last European person believed to know everything at least at the level of scientific knowledge in Europe was Sir Francis Bacon (1561–1626). In Richard Hamming's seminal work, published 30 years ago, he described many aspects that he found were necessary to scientific success over his long experience conducting observations, explorations and interviews with fellow scientists, especially Nobel Prize laureates (Kaiser 2010). Hamming also stated that scientific knowledge doubles every 17 years, but 30 years later (in 2018) it is generally accepted that the pace is significantly faster and that different scientific fields have different rates of development. Hence, it is difficult to define a uniform rate of knowledge growth, although it is currently (arbitrarily) set to much less than 12 months. This exponential growth rate is expected to continue in the future and become commonplace for future scientists. One great challenge is how to structure old and new generated information in a way that allows scientists to determine which information is relevant and retain only that. This has led to ongoing differentiation of the field of science into sub-disciplines that have become progressively disconnected from each other, leading to intense specialisation in pursuit of success.

What is scientific success? Being highly cited, publishing many books or high number of papers with low citation frequency, maybe patents that are of interest to industry? Being accepted by students and adored by Ph.D. students as a good mentor? All layers of the relevant information concerning a published paper, such as the underlying value of a study or the extent of improvement after solving the problem at hand, cannot be captured by citation-based metrics (Hutchins *et al*. 2016). Also, this approach cannot be used to appropriately describe the applied research and target a narrower audience, such as engineers or clinicians. Nevertheless, it is generally accepted that the highly cited papers are more influential than average, while uncited articles exert a marginal influence (if any) on their respective scientific fields (Hutchins *et al*. 2016). However, a few of the most influential works, such as Einstein's paper on the theory of relativity, have so quickly become common knowledge and included in general books that no one cites them anymore.

In order to place oneself within the scientific community, one needs to understand the topology of the publishing industry. If you were to print out just the first page of every item indexed in the Web of Science as of 2014, the stack of paper would extend to the top of Mt Kilimanjaro (5895 m), an impressive, monumental pile of paper. Only the top metre and a half of that stack would have received 1000 citations or more, and just a centimetre and a half would have been cited more than 10 000 times. The top 100 papers are cited more than 12 000 times, besting some of the most recognisable scientific discoveries in history (Van Noorden, Maher and Nuzzo 2014), while 74% of published papers either are never cited or attract fewer than a dozen citations. There are other metrics, but citation index is one of the most commonly used ones, and from the perspective of applications, successful grant writing and financing your own research, being highly cited is definitely a benefit.

In order to become highly cited, there are two strategies, one can either travel alone and quickly or travel with others and go far. An approximate example of the former is the ‘unemployed gentleman scholar’ Edgar (2014) who noted that career paths are hardly planned or plannable, so you cannot really follow anyone, except to go where there is no path. On the other hand, however, one could look at the careers of accomplished scientists (Hamming 1997; Kaiser 2010) who made astonishing achievements through institutional groups and large collaborations. On any path similar to the two proposed here, knowledgeable people pay great attention to and invest large amounts of energy in how things are done (e.g. problem selection, evaluation of research methods, making conclusions and determining the validity of conclusions).

Two dozen basic characteristics that are necessary for success in science were proposed in Hamming's (1987, published in Kaiser 2010) timeless essay and were collated in this paper for readers interested in them. In addition, it is high time and worthwhile to acknowledge and put into perspective all critically discussed ideas and practical examples in ‘Ten Simple Rules for Doing Your Best Research, According to Hamming’ (Erren *et al*. 2007), ‘Ten Simple Rules for Lifelong Learning, According to Hamming’ (Erren *et al*. 2015), ‘How to Surf Today's Information Tsunami: On the Craft of Effective Reading’ (Erren, Cullen and Erren 2009), ‘On the Process of Becoming a Great Scientist’ (Giddings 2008), ‘Ten Simple Rules for Writing Research Papers’ (Zhang 2014), ‘Ten Simple Rules for Creating a Good Data Management Plan’ (Michener 2015), ‘Ten Simple Rules for Reducing Overoptimistic Reporting in Methodological Computational Research’ (Boulesteix 2015), ‘Ten Simple Rules for Better Figures’ (Rougier, Droettboom and Bourne 2014), ‘Ten Simple Rules for Effective Computational Research’ (Osborne *et al*. 2014) and others. This paper aims to present an extended review coupled with a brief historical summary and metadata analysis of publication network data and to serve as a starting point for future discussions within the scientific community.

## TODAY: THE VAST MAJORITY OF ALL SCIENTISTS ARE ALIVE

One could falsely think that there is no room for error in science and that we are striving for perfection. For those who actually do lab or computer work themselves, Pareto's rule—that 80% of the tasks should be done correctly in 20% of the time—is one of the best gauges for the first trial. This means that hard work must be applied smartly in order to produce meaningful results that support scientific advancement (and will be cited as well). For differences between smart work and non-smart work, please see Cipolla's (2011) book *The Basic Laws of Human Stupidity*, which rather efficiently presents the two principles that guide human decision-making.

It is well established that interdisciplinary collaborations often offer opportunities for scientific breakthroughs. This is exciting, and it at least partly explains the great rate of scientific advancements as the vast majority (>95%) of all scientists who have ever lived are alive today. In addition, this population is rather young, either in the process of obtaining their degrees or performing research at early stages of their careers. While education is more interested in when and why to do certain things, training involves how to do things (Hamming, 1997). When in training, one needs to consider and think about factors that they were not inclined to think about before. It is very productive to clear misconceptions when they are being formed as this can simplify and advance matters.

## LEGACIES: THE EXAMPLE OF MEDICAL MICROBIOLOGY

Let us use medical microbiology as an example of a best-case scenario as it has been overwhelmingly successful in helping to minimise many of most deadly and contagious diseases over the last 150 years. The history of humankind and Earth would be completely different if the most medically relevant microbes and viruses were as difficult to cultivate under laboratory conditions as their counterparts in the environment (i.e. 99.9% of all microbes and viruses). Hence, it is relatively straightforward (but not easy; we do not underestimate the complexity of the problem nor the amount of work and intellectual efforts) to isolate various causative agents in pure culture and then devise antidotes, antibiotics and vaccination. However, most of these microbial agents were subjected to culture, while most microbes in nature have not been. The success of the principle of ‘one agent–one disease’ has inadvertently led to substantial delays in development of microbiology; for over a century, many studies have attempted to pursue and repeat a ‘winning’ approach, such as cultivation techniques. The cycle of hype and then lack of results have opened the door to a centuries-old realisation that the number of cells on a plate or in a tube does not correspond to the number of directly counted cells. Also, the diversity on plates does not match that observed in nature, regardless of which estimator was used initially. This eventually led to the adoption of ideas from other fields, such as microfluidics, physical chemistry, ecological theory, bioinformatics and applied statistics.

The historical platform of the dominance of the bottom-up approaches in microbiology since its conception hence determined much of what it was to follow from van Leeuwenhoek microscopy, Koch postulates, Pasteur vaccination and fermentation developments, and work of many other splendid scientists. The rather simple means available at that time coupled to high level of ingenuity and insight paved the road of constant methodological developments; however, due to the simplicity of approaches, only the idea of bottom-up type experiments seemed effective mode of research on microbial complex systems. In essence, the successes of medical microbiology have left tremendous legacy in the way minds of researchers operated in the 20th century and it extends to these days.

## LESSONS: MICROBIAL ECOLOGY

The introduction of molecular techniques to microbial systems biology, such as microbial ecology, has led scientists from a variety of fields to maintain many decade-long arguments on the extent of distortion of the microbial world by conventional or recently developed (and more complex) cultivation techniques. Although researchers have made efforts to improve cultivation approaches and the extent of distortion is debatable, the fact that it was prohibitively excessive paved the way for the development and application of rather novel approaches in real time (Passoli *et al*. 2019). The root cause of this distortion stems from the measurable discrepancy between habitat conditions successfully cultivated in the laboratory on one hand and survival, co-metabolism or growth in nature on the other. The belief that a human-made environment cannot attain the necessary environmental, temporal, chemical, physical and genetic complexity has diminished over the decades since great microbiologists, such as Winogradsky, Beyerinck, Perfiliev and Kluyver, first acknowledged that most complex natural habitats cannot be reproduced using an agar plate, roll tube or shake flask. Once (a)biotic conditions diverge and become too different (i.e. not complex or dynamic enough), microbes from those complex habitats cannot occupy their ecological niche, maintain their metabolic activities or maintain acceptable thermodynamic balance, and consequently cannot recover from cultivation attempts, regardless of their frequency. From this, it follows that the environmental conditions of particular microbial communities under investigation need to be recognised before hotspots in the ecological niche, in line with the top-down research approach, in order to guide subsequent cultivation attempts and avoid labelling microbes as ‘uncultivable’ even though they effectively grow and evolve in their natural environment. The realisation that organisms (or their essential co-cultures, such as syntrophs, basic cultivation units) need to have a complement of their ecological niche in order to yield a culture improved our understanding of microbes’ thermodynamic needs. This, in turn, led us to devise and re-engineer a hospitable cultivation environment with the right dynamic supply of all necessary substrates and nutrients (through provision or production by other microbes or hosts), eliminating products (via mass transport and dilution or by other microorganisms) and controlling environmental parameters (pH, temperature, redox potential, electrical conductivity, alkalinity, etc.). The currently available microbiological cultivation techniques of various provenances are not equipped or designed to effectively deal with such complexity. It is possible that future high-throughput techniques can deliver the necessary complexity with automated and controlled approaches (Op den Camp, Jetten and Strous 2007). Microbiologists (and microbial ecologists) should remember when doing cultivation work that their laboratory systems are models and cannot reflect ‘real’ conditions.

## MICROBIAL COMPLEXITY: MICROBIAL ECOLOGY, SYSTEMS BIOLOGY AND SYSTEMS MEDICINE

Complex systems arise and evolve through self-organisation, a dynamic process of forming nontrivial macroscopic structures and behaviours over time. As a result, systems are neither completely regular nor random and give rise to emergent behaviour at macroscopic scales that is difficult to explain from microscopic properties, despite the relationship between the properties of a system at the micro- and macroscopic scales (Sayama 2015). Large-scale behaviours can emerge from the correlated or dependent behaviour of individual small-scale components. Dependent behaviour among system components results in overlapping or shared information. A system's structure is revealed by the sharing of information across the system's dependencies, each of which is associated with a certain scale (Allen, Stacey and Bar-Yam 2017).

**Figure 1.**
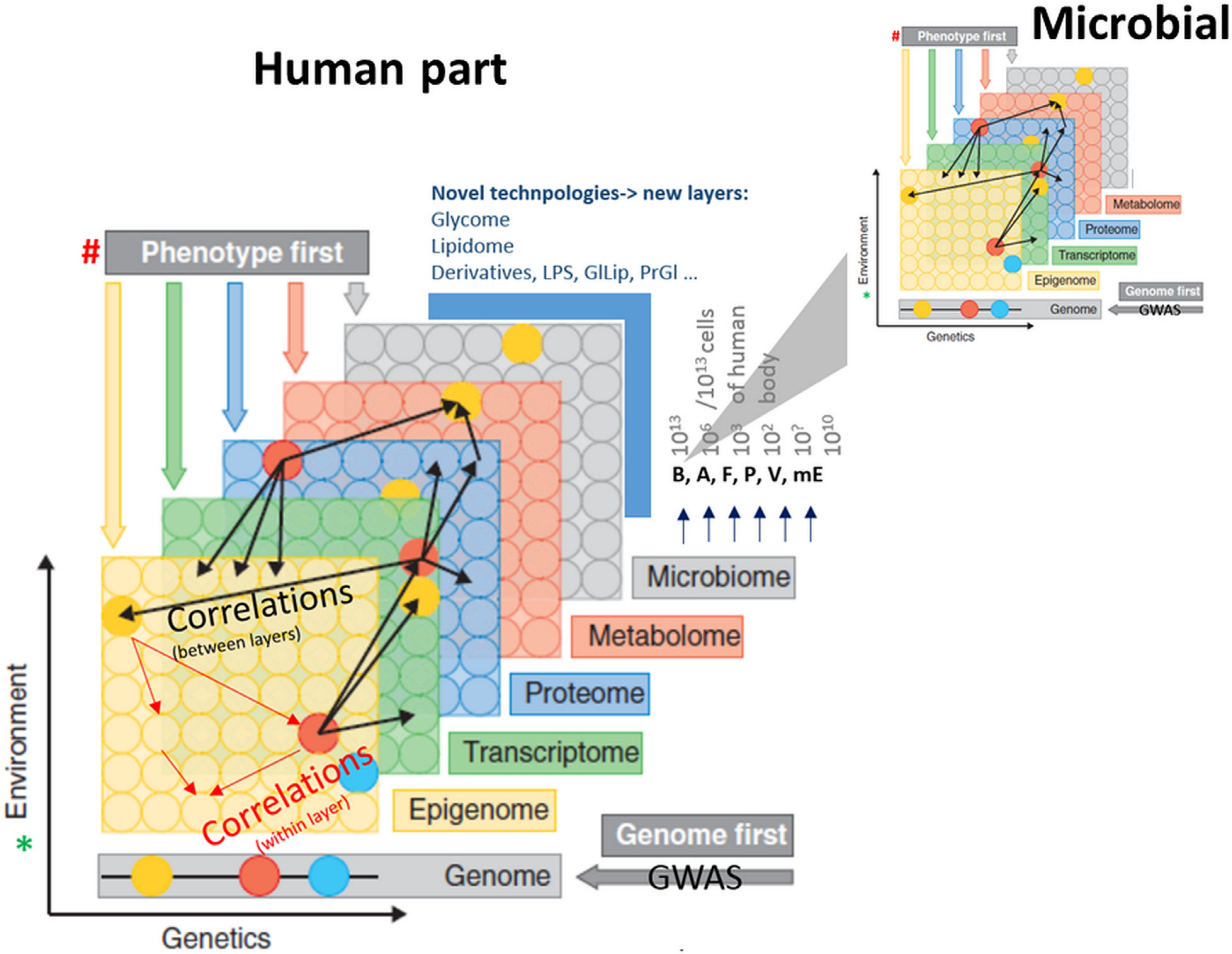
Example of the multilevel and multi-omic layers of information that are being integrated in current multi-scale approaches to the integration of microbiological information into natural systems (reproduced with permission and modified; Hasin, Seldin and Lusis 2017). Circles represent the entire pool of molecules detected in various ‘omic’ data layers. Genetic regulations and environments are embedded within all data layers, except the genome (GWAS) layer, and can affect each individual molecule to a different extent. The potential interactions or correlations between molecules detected within one layer or between different layers are represented by thin red and black arrows, respectively. As an example of the conceptual framework for consolidating multi-omic data to understand the function of the system, the gene in a genome (blue circle) is epigenetically regulated (red circle) and controls multiple transcription targets correlated with multiple proteins that generate metabolites, which can have a greater influence on the microbiome layer as well. The three firsts (i.e. the genome first, the phenotype first and the environment first) imply a starting point: the associated locus versus any other layer versus environmental perturbations (i.e. thermodynamic boundaries within which the system routinely operates). GWAS: genome-wide association studies; B: bacteria; A: archaea; F: fungi; P: protozoa; V: viruses; mE: mobile elements; LPS - Lipopolysaccharides; GlLip - Glycolipids; PrGl - proteoglycans.

A multi-scale structure is one of the key features of complex biological systems (Fig. 1). Such structures involve hierarchical organisation of the smaller building blocks in multicellular organisms, such as tissues, fibres, proteins, amino acid motifs, DNA, RNA, lipids, various glycosylated residues and their mixed forms, such as micelles (Cordero and Datta 2016). Their interaction yields complex patterns and oscillations in gene expression, metabolism and energy flow over the systems of dense microbial communities and multicellular organisms. In addition, biological systems have another hierarchical axis on which complexity increases from individual organisms to populations, communities and meta-communities. Therefore, it is essential to consider whether all macroscopically evident properties, such as the functions of microbial ecosystems (landscapes or host-associated ecosystems), can be simply reduced to measurements of the functions of their components, without considering how these components interact (Cordero and Datta 2016). On the other hand, our understanding of living systems is based upon technology-driven assessment of the status of the genomes, transcriptomes, proteomes, metabolomes of single cells, organisms or cell cultures in specific environmental settings with thermodynamic boundaries. At the level of integration across scales and complex microbial communities, this phenomenon resulted in the current state of the ‘omics’ field (i.e. metagenomic, metatranscriptomic, metaproteomic and metametabolomic datasets), and it is used to infer the associations between the interacting genomes of many microbial species over time and space (Fig. 1). Such associations have been shown to participate in many competing, supporting, commensal, positive and negative interactions and to generate feedback or feed-forward regulatory loops.

This compound (sub)system generates a meta-phenotype of a microbial community that is actually interacting with its host (e.g. the intestinal tract, lungs or rhizosphere). The effects of this meta-microbial phenotype increase over time and space, leading to next-level integration of the two subsystems (i.e. the host and meta-microbial phenotype) into a meta-phenotype of the system (Fig. 1). From this perspective, inclusion of a microbiome when performing system-wide integration of information over multiple scales is inevitable and amenable to top-down modelling (Hasin, Seldin and Lusis 2017), allowing microbiology to receive the benefits of other fields of science, such as machine learning, microfluidics and statistical bioinformatics.

## EMERGING PATTERNS: RECENT DEVELOPMENTS IN TOP-DOWN RESEARCH TOPICS

**Figure 2.**
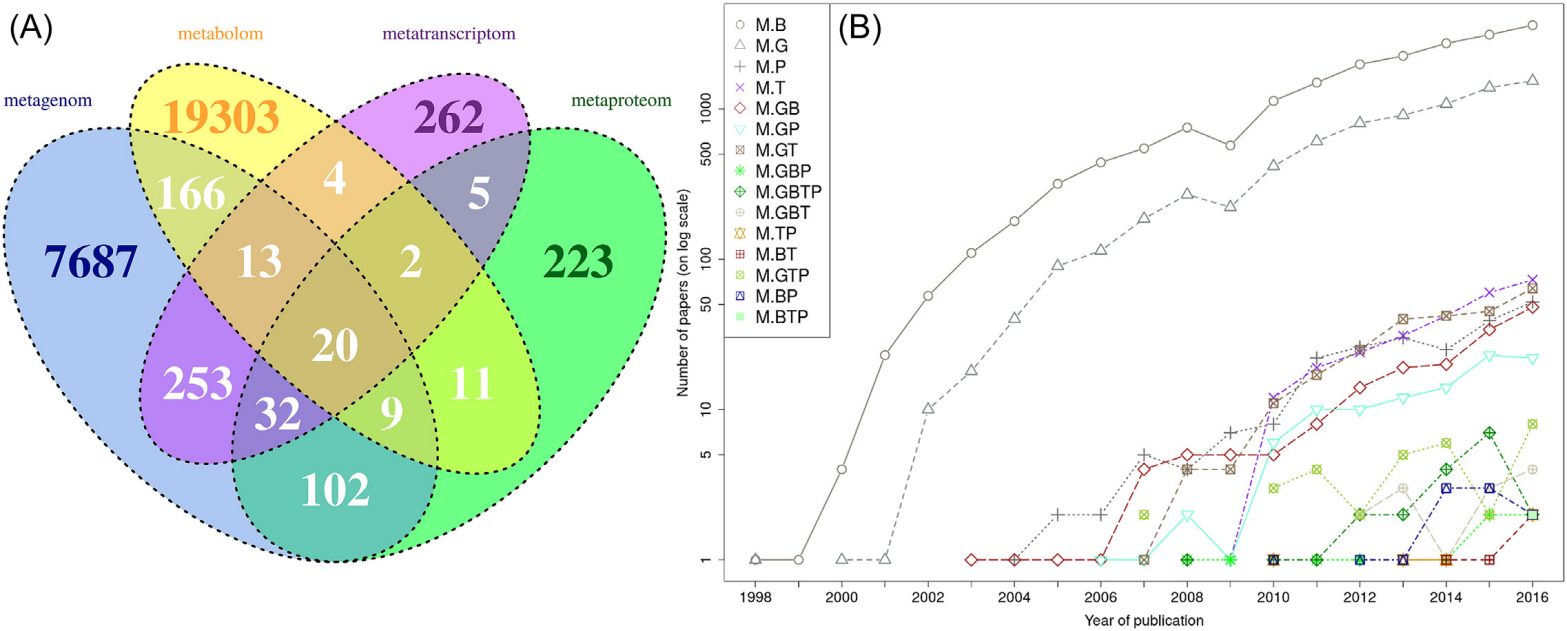
**(A)** Venn diagram of publications sharing basic keywords. **(B)** Production rate of papers dealing with one or more sub-disciplines. The major four (top-down) meta-versions of disciplines within microbiology: M.G, M.T, M.P and M.B.

This study assesses the trend among top-down approaches (i.e. the potential for collaborative undertakings) by scientometric analysis of citations and collaboration networks across the major four (top-down) meta versions of microbiology disciplines: metagenomics (M.G), metatranscriptomics (M.T), metaproteomics (M.P) and metabolomics (M.B). From a scientometric perspective, a network of citations among scientific papers or patents represents the cognitive network (i.e. the flow of ideas), while a collaboration (i.e. co-authorship) network depicts the social network among the authors of published scientific contributions. The extent of overlap between various disciplines in the citation network, bibliographic coupling network and authorship networks was explored (for technical details on this analysis, see the section on the materials, methods and data). Based on the available published papers, the largest and oldest ‘omics’ sub-discipline is M.B (*n*_metabolomics _= 19 522), followed by M.G (*n*_metagenomics _= 8281). M.T and M.P had roughly 30 times fewer papers (*n*_metatranscriptomics _= 591, *n*_metaproteomics _= 404). The extent of overlap between various topics seems to be rather small (Fig. 2A). The rate of publishing (Fig. 2B) reveals the existence of three major production groups. The first group, which publishes hundreds of papers, includes M.B and M.G, which emerged around the year 2000. The second group, which publishes tens of papers per year, contains papers on M.T, M.P and pairwise combinations of subtopics (M.GP, M.GT, M.GB). This group appeared 10 years after M.B and M.G emerged. The number of published papers in this group grew slightly slower than in the first group but generally followed the same trend. The third group, which publishes just a couple of papers per year, contains all other subtopics, including the most complex, M.GTPB (i.e. system-wide integration), which is still in its infancy. However, it is going to be of great interest to observe how this group's trends are going to develop in the future.

## OVERLAP OF IDEAS AND PERSONNEL: THE FIRST SIGNS OF TRANS-DOMAIN RESEARCH ACTIVITIES

**Figure 3.**
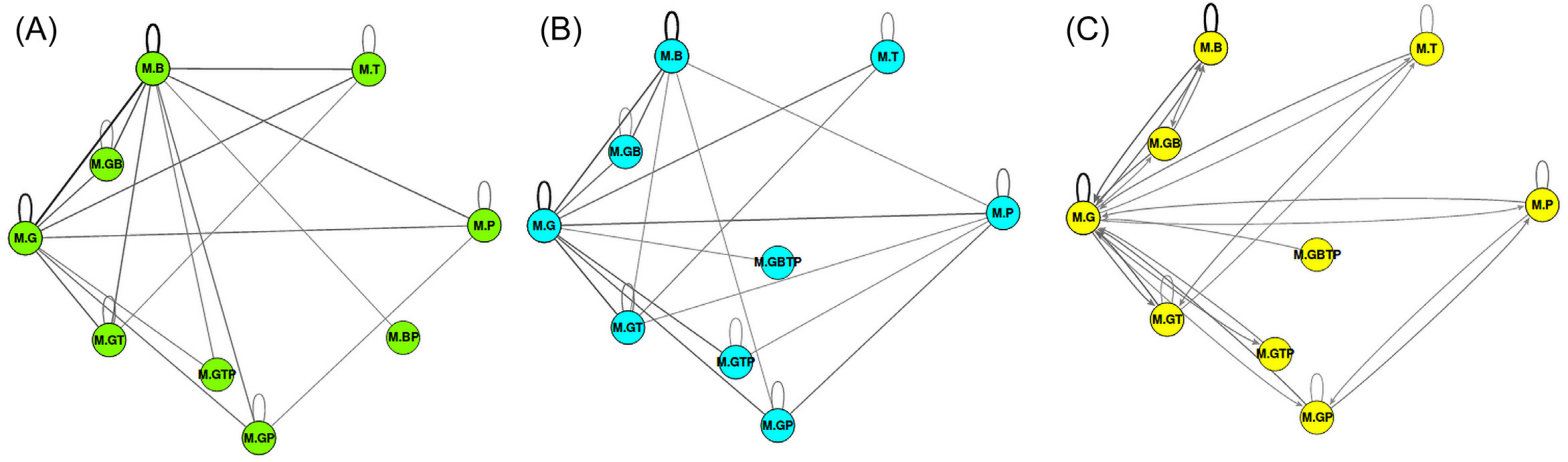
**(A)** Papers on select subtopics authored by the same authors, **(B)** bibliographic coupling (i.e. co-cited papers) and **(C)** citation of meta-omics papers on select subtopics. Loops in the networks represent connections between papers within a selected subtopic. All the values of ties within the networks are log-transformed and max-normalised. Only ties with values higher than 0.35 are presented. Unconnected subtopics (i.e. those for which ties were cut) are hidden.

The overview of the three analysed networks presented in Fig. 3 shows the natural dominance of M.B and M.G, which emerged before all other subtopics. Beginning the analysis with the authorship network (Fig. 3A), strong connections between clean M.G, M.B, M.T and M.P subtopics can be observed. This indicates that, for example, authors of M.G papers also wrote papers in M.P. There is only one missing connection within the clean subtopic group, a connection between M.T and M.P, which reveals the social gap between these two subtopics. Relatively strong connections are present among pairwise combinations of subtopics from the second group, as indicated in Fig. 2B. It also appears that most authors remain within a single subtopic, as indicated by the loops above clean subtopics and larger (M.GB, M.GT, M.GP) combinations. This suggests the existence of subtopic authorship communities.

The coupling network includes papers that are cited by researchers focusing on two connected subtopics (see Fig. 3B), while a ‘directed’ citation network (Fig. 3C) includes citations between papers on connected subtopics. The first of these two networks can be interpreted as a common cognitive foundation for connected subtopics, while the second one reflects the flow of ideas between connected subtopics. In both networks, the most central position is occupied by the clean M.G subtopic, although it is smaller in all aspects and slightly younger than M.B. Generally, the coupling and citation networks are very similar and reveal some interesting characteristics of subtopics’ interactions. The weights of reciprocal ties in the citation network are slightly higher for clean subtopics, with one exception: papers on M.P cite those on M.GTP more often than M.GTP papers cite those on M.P (because of the cut-off for lower values, this is not visible in Fig. 3C). By adopting the top-down approach for analysis of the scientific publication data, we were able to illustrate general patterns in multi-scale field development over time that could not be detected otherwise. This simple example illustrates how examining existing information and data can improve our understanding of global trends in the development of science and may have the potential to foster new research directions.

## CURRENT STATE OF AFFAIRS: THE NEED FOR META-ANALYSES TO LEVERAGE BIASES

The analysis presented in this study can be taken also as an example of top-down approach. It is basically a meta-analysis, instead of a traditional narrative review, and hence represents a more objective and informative way of summarising the research question (Nakagawa *et al*. 2017). The majority of microbiologists have probably never attempted to conduct a meta-analysis, but we hope to be proven wrong in the future. A meta-analysis, if conducted correctly, can provide not only quantitative information (i.e. the distribution of publishing rates between fields) but also qualitative information (i.e. the dominant research gaps), hence providing an unbiased overview of the field. Due to the immense (and increasing) availability of published data and papers, microbiology training (as well as training for other disciplines) should, in our opinion, include the meta-analytic approach as part of researchers’ and students’ standard toolbox for critically evaluating and interpreting their results and the results of publicly available literature or larger corpora of published information. Currently, one of the most common remarks in seminars is that there are many contradictions between papers. Meta-analyses help to overcome various barriers embedded in scientific thinking, which may be intellectual (i.e. confirmation bias, pattern seeking, belief bias) or practical (i.e. inefficiency of factorial design, parsimony attraction, publication bias), via creative thinking, blind analysis or collaboration with researchers with opposing views (Bettini *et al*. 2016) or statistical modelling based on the design of experiments (Perkel 2016). Our understanding of biological systems relies most heavily on the quality and reproducibility of basic science data (Ioannidis 2005; Prinz, Schlange and Asadullah 2011; Nature Chemical Biology 2013), which in turn impacts the clinical development of the field (Arrowsmith 2011; Asher 2011; Kleikers *et al*. 2015). However, it is impossible to assemble all available information and obtain fair insights for the following reasons:

- Citation bias, which leads less-referenced sources to be less likely to be included in further studies or meta-analyses.
- Dissemination bias, which leads the results of the study to be unevenly reported (i.e. the author selectively emphasises one portion of the results over another).
- Grey literature bias, which leads harder-to-find literature to be ignored (e.g. government reports, unpublished clinical studies).
- Language bias, which leads foreign-language studies to be excluded from analyses or the language issues of non-native speakers to factor into researchers’ decision to include them in analyses.
- Media attention bias, which leads studies to be included in meta-analyses because of media attention.
- Outcome-reporting bias, which leads positive outcomes to be more likely to be included in a meta-analysis than negative outcomes or negative outcomes to be misrepresented as positive ones.
- Time-lag bias, which leads studies with significant results to have a shorter median time to publication (4.7 years) and those with non-significant results to have a longer median time (8.0 years; Song *et al*. 2010; Hawkes 2013).
- High effect size anomalies, which leads studies that report relatively high effect sizes to be more likely to be published than studies that report lower effect sizes.

Meta-analyses are not omnipotent (always able to leverage the biases in published studies), as any bias in the published literature will also be taken up during the process of selecting eligible papers for the meta-analysis (Borenstein *et al*. 2011). Without additional care, any meta-analysis or literature review based on naively assembled published data is going to be biased. If reports not published in SCI journals cannot be included, the researchers should check their data distribution using an approach such as funnel plot asymmetry, in which the *Y*-axis represents precision (i.e. the standard error) and the *X*-axis represents the effect size of individual studies (i.e. the standard difference in means). Ideally, the errors in reported parameters that can be observed in a meta-analysis should be normally distributed. Using trim and fill analyses, one can explore and adjust for possible missing studies within the published data used in the meta-analysis to estimate the quality of the meta-analysis. If errors are normally distributed, a low number of studies would generate less information, wider confidence intervals and less powerful tests, but the effect size will not be systematically affected. On the other hand, if errors are not distributed normally, then the missing studies are systematically different from those used in the meta-analysis and the sample of studies is biased. Therefore, it is of vital importance to address whether there is any evidence of bias, whether the observed effect is an artefact of bias, how much of an impact bias has via imputation techniques and, finally, whether it has any biological meaning (Borenstein *et al*. 2011; Kleikers *et al*. 2015).

The increasing rate of publications and production of data, algorithms and statistical approaches has led some leading journals to reconsider their publishing approaches or suggest that drastic changes are underway due to the recently identified biases of (ix) data availability (Nature Methods 2016b), (x) algorithm availability (Nature Methods 2016a) and (xi) database bias, according to which the availability of machine-readable and curated data enhances the visibility and reuse of published or publicly released data, leading to increased citations or original publications. To gain access to certain datasets and algorithms for re-analysis of the data in the broad context of a meta-analysis, researchers need to contact editors to register the unavailability of materials from authors when restrictions to access were not mentioned in the paper. As an example, the vast majority of potential results were missed due to the use of outdated databases in up to two-thirds of about 3900 recent publications. These studies also missed and underestimated the functional significance of their own results, which had a negative impact on the hypothesis generation process and led to misinformed and misleading prospective studies (Wadi *et al*. 2016). Validation experiments performed by re-analysing existing data through meta-analyses have yet to be performed to ameliorate this. Similar to the way in which microbiologists must keep in mind that microbial cultivation systems represent lab models, one should remember that meta-analyses do reflect models but are built on a large number of cases and thus yield more generalised and less biased composite models than those built within a single study.

## FUTURE: INTERACTION BETWEEN DISCIPLINES CAN BRING LOOSE ENDS TOGETHER

We challenge Hamming's view of the continuous crumbling of science into highly specialised subfields and instead propose learning from the positive outcomes of crossover of relevant knowledge from different subfields, which yields additional insights beyond a high degree of specialisation within a particular subfield (Fig. 2A). We also challenge Hamming's 30-year-old idea that researchers should shift to novel fields in order to retain spirit and originality. Although broadly correct, we naively feel that trans-domain interdisciplinarity is more likely to be the dominant paradigm in the future that provides necessary drive, novel ideas, throughput and technologies. Instead of further specialising in one field and then moving to another field for additional specialisation, tightly collaborating consortia headed by multiple top researchers can have novel ideas and realisations through high-end science at the same time due to the synergistic, stimulating effects of other highly skilled participants in the consortium. In our experience, taking excellence (embedded in ideas and personnel) from one field to another can improve the other field, leading to unanticipated hybridisation of knowledge. Multi-furcating branches of currently unrelated subfields can then be combined into one coherent field that performs research at different scales and brings together many different perspectives on the same problem. Instead of repeating the historical calls for ‘more microbiology’, we believe that more cross-fertilisation among fields is needed to advance the field of microbiology, as similar trends are already happening, according to the re-analysis of published literature performed in this study.

## LAST BUT NOT LEAST: TECHNOLOGY AND ARTIFICIAL INTELLIGENCE ARE THE FUTURE

**Figure 4.**
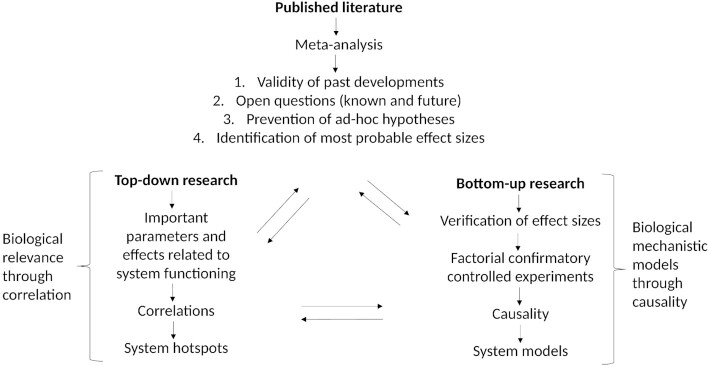
Missing opportunities and balance of the three basic approaches—meta-analysis, the top-down approach and the bottom-up approach—as sources of information to leverage the understanding of systems.

Most techniques that microbiology used until 1990 were derived from the field of medicine. There is not much difference 30 years later, except the sources from which tools are adopted are more diverse. Modern microbiologists’ toolboxes are being continuously amended with tools from medicine, chemistry, chemometrics, quality assurance, biotechnology, mathematics, physics, design of experiments, engineering, IT, many statistical fields, power analysis, meta-analysis, bioethics, experiment replication, data analysis, storage and modelling, and bioinformatics. Thus, it is not surprising that technological advances have transformed microbiology in the past 15 years due to the sequencing revolution, the evolution of statistical theory and software (e.g. R), computational resources, miniaturisation, high throughput and the adoption of more rigorous quality standards. Multi-scale microbiology can be considered a largely technology-, data- and statistics-driven discipline with numerous subfields at different scales (Fig. 4).

It is worth considering what would happen if microbiologists were ready to embrace the idea that they are missing opportunities because of scattered datasets, an inability to perform meta-analyses, a lack of statistics, a lack of design of experiments, a lack of bioinformatics, outdated databases, a lack of understanding of the history of the field and aversion to new technologies. Here, we argue that the high-throughput top-down multi-omic approaches are amenable to direct mathematical modelling, which is one of the most underused parts of the microbiology toolbox despite its high promise for identifying generic mechanisms, statistical rules, time frames of microbial assemblages and, hence, the important parts needed to develop a quantitative theory of microbiomes (Goldford *et al*. 2018). Technologically driven multi-scale microbiology is already a reality, and it is increasingly coupled with automation, modelling and artificial intelligence, being called systems biology, systems medicine, multi-scale medicine and systems ecology, as part of big data science (Zanin *et al*. 2017). What will be used to deliver it is entirely up to us. We acknowledge that it is smart to stand on the shoulders of giants, traveling fast and with others.

## MATERIALS, METHODS AND DATA

The analysed data were gathered from the Thomson Reuters Web of Science (WoS) database. Complete information on papers (i.e. bibliographic items), including paper title, journal name, abstract, keywords, authors, year of publication, cited references, number of citations and some other characteristics, is accessible on the WoS. Thus, from the WoS, we obtained complete information about all published papers on a given topic: ‘metabolom*’ or ‘metagenom*’ or ‘metaproteom*’ or ‘metatranscriptom*’. The WoS service considers a paper to concern a certain topic if the search term is present within the title, abstract, author keywords or WoS keywords ‘plus’ the bibliographic items. In April 2018, we obtained information on 28 654 papers published from 1900 until the end of 2017. It is worth mentioning that the number of papers on a given topic increases rapidly each day. Just for example, the number of papers we identified when we ran the same query in August 2016 was 25 782, meaning that more than 500 papers were published on the topics each month. The obtained data were treated with the WoS2Pajek programme (Batagelj 2016), which transforms data from the WoS into a collection of compatible networks and vectors with node attributes (e.g. the year of publication).

In the section on the overlap of ideas and personnel, we analysed the collaboration network derived from authorship data, the citation network and the bibliographic coupling network derived from the citation network (see Batagelj, Ferligoj and Squazzoni 2017). Detailed information about the network transformations can be found on the WoS2Pajek webpage. For the visualisations, which were prepared in Pajek (De Nooy, Mrvar and Batagelj 2018) and presented in Fig. 3, the values of connections within each of the networks were log-transformed (to reduce skewness), divided by the maximum value (normalised to 0–1) and cut off at the 0.35 level.

The papers obtained through the WoS system could concern only one of the four meta-omics topics or a combination of two, three or all four. Overlaps in topic can be represented with a Venn diagram (Fig. 2A). In the analysis, 15 possible combinations of the four topics are considered subtopics. The units of citation, coupling and authorship networks (i.e. papers and authors) are merged according to their affiliation to selected subtopics. The obtained groups are treated as units of analysis.

## SUMMARY, CONCLUSIONS, OUTLOOK AND FUTURE PROSPECTS

In this work, the position of contemporary microbiology was considered from the perspective of scientific success, and a list of historical points and lessons learned from the fields of medical microbiology, microbial ecology and systems biology was presented. In addition, the emerging patterns of development of top-down research topics over time and overlapping ideas and personnel, as the first signs of trans-domain research activities in the fields of metagenomics, metaproteomics, metatranscriptomics and metabolomics, were explored through analysis of publication networks of 28 654 papers with the Pajek programme. As a result, the current state of affairs was defined and the need for meta-analyses to leverage publication biases in the field of microbiology was proposed to be a very important emerging field of microbiology research, especially since microbiology is increasingly dealing with multi-scale systems. We argue that, to advance the field of microbiology, there is a need for cross-fertilisation with other fields/disciplines instead of ‘more microbiology’. In this manuscript, the term ‘cross-fertilisation’ does not distinguish between personal scientific affairs (e.g. boosting of scientists’ careers or publication success) on one hand and general scientific affairs (e.g. the integrity of data generation and data publication) on the other, as we believe that these are tightly linked, representing two sides of the same coin. The reader is directed to consider how novel technologies, the introduction of big data approaches and artificial intelligence have transformed microbiology into a multi-scale field that has shifted away from its history of mostly manual work and towards a largely technology-, data- and statistics-driven discipline that is being progressively coupled with automation and modelling.

## DISCLAIMER

The references cited in this paper are not an exhaustive list of publications, but are provided to serve as a starting point and guidance.

## Supplementary Material

fnz159_Supplemental_File
